# Visual Contextual Effects of Orientation, Contrast, Flicker, and Luminance: All Are Affected by Normal Aging

**DOI:** 10.3389/fnagi.2016.00079

**Published:** 2016-04-18

**Authors:** Bao N. Nguyen, Allison M. McKendrick

**Affiliations:** Department of Optometry and Vision Sciences, University of Melbourne, ParkvilleVIC, Australia

**Keywords:** surround suppression, visual cortex, center–surround, contextual effects, aging

## Abstract

The perception of a visual stimulus can be markedly altered by spatial interactions between the stimulus and its surround. For example, a grating stimulus appears lower in contrast when surrounded by a similar pattern of higher contrast: a phenomenon known as surround suppression of perceived contrast. Such center–surround interactions in visual perception are numerous and arise from both cortical and pre-cortical neural circuitry. For example, perceptual surround suppression of luminance and flicker are predominantly mediated pre-cortically, whereas contrast and orientation suppression have strong cortical contributions. Here, we compare the perception of older and younger observers on a battery of tasks designed to assess such visual contextual effects. For all visual dimensions tested (luminance, flicker, contrast, and orientation), on average the older adults showed greater suppression of central targets than the younger adult group. The increase in suppression was consistent in magnitude across all tasks, suggesting that normal aging produces a generalized, non-specific alteration to contextual processing in vision.

## Introduction

The perceived properties of a visual stimulus can be markedly affected by the spatial context in which it is presented. For example, the perceived contrast of a textured patch or grating appears to be of lower contrast when surrounded by a high-contrast pattern than when it is presented on a uniform field (Chubb et al., 1989; Cannon and Fullenkamp, 1991; Snowden and Hammett, 1998; Xing and Heeger, 2000). Visual neuronal responses throughout the primate visual pathways show similar contextual behavior, where the spike output from the central receptive field depends on the stimulus conditions presented to the near and far surround regions of the extraclassical receptive field (for example, see Shushruth et al., 2013). Neurophysiologically, such effects arise from combinations of lateral, feedforward, and feedback inhibition (reviewed by Angelucci and Bressloff, 2006; Nurminen and Angelucci, 2014), and at multiple stages of the visual system, thereby providing the neuronal architecture that may support the human perceptual observations.

Of specific interest to our study is the fact that some perceptual effects of background context are considered to arise from pre-cortical neural circuits, whereas others are more consistent with cortical neuronal properties. Two effects that have been linked to pre-cortical processing are suppressive effects on the perception of (a) luminance and (b) flicker. For example, a gray patch appears darker when placed on a bright background, and vice versa (Heinemann, 1955). Models to explain such luminance suppression are based on pre-cortical mechanisms of lateral inhibition in the retina and lateral geniculate nucleus (LGN; Valberg et al., 1985; Creutzfeldt et al., 1991). When the luminance of the center and surround components of a homogenous stimulus are flickered at different temporal phases, the center perceptually segments from the background (Kelly, 1969); however, the perceived strength of flicker at the center is reduced (Kremers et al., 2004). The physiological basis for flicker suppression is already present at a pre-cortical level. Response amplitudes in LGN cells of monkeys and the perceived flicker strength of the center stimulus in humans are both modulated in qualitatively the same way by the phase difference between the center and surround components (Kremers et al., 2004). Furthermore, the size dependence of flicker suppression can be described by space constants that are commensurate with the spatial extent of receptive fields of macaque and marmoset LGN cells (Kremers and Rimmele, 2007). More recently, comparisons of dichoptic (presentation of the center and surround stimuli to separate eyes, thereby invoking cortical mechanisms to perceptually combine) and monoptic conditions (presentation of the center and surround stimuli to the same eye, which enables combination of the stimuli at both cortical and pre-cortical levels) confirm that modulation of perceived flicker strength involves substantial pre-cortical involvement, in addition to cortical contributions (D’Antona et al., 2011; Teixeira et al., 2014).

Pre-cortical and cortical mechanisms are also implicated in the contextual processing of contrast. Perceptually, the contrast of a pattern appears lower when embedded in a high-contrast background (Chubb et al., 1989; Cannon and Fullenkamp, 1991; Snowden and Hammett, 1998; Xing and Heeger, 2000). Functional magnetic resonance imaging (fMRI) in human observers demonstrates response suppression in primary visual cortex (V1) while observers are visualizing stimuli that evoke a perceptual contrast suppressive effect (Zenger-Landolt and Heeger, 2003), while convergent neurophysiological evidence points to additional pre-cortical and extrastriate cortical contributions. For example, depending on the contrast of the center stimulus, primate neuronal spatiotemporal tuning of suppression is broadband and monocularly driven when the center contrast is low (implying processing at the LGN and input layers of V1) whereas beyond the input layers of V1, suppressive effects are narrowly tuned, binocularly driven, and prominent when high-contrast stimuli drive the central neuronal response (Webb et al., 2005). Other contextual effects that are considered to be predominantly cortically mediated are those that involve orientation judgments – for example the tilt illusion, whereby the perceived orientation of a center target tends to be shifted in the opposite way (‘repulsive’ tilt illusion, O’Toole and Wenderoth, 1977) from the orientation of the surrounding pattern (reviewed by Clifford, 2014). To explain this phenomenon, orientation-selective neurons that respond to the surround orientation are thought to suppress (via lateral inhibition), similarly-tuned neurons that respond to the center target. When the center and surround orientations differ slightly (e.g., by 15–20°), the overall population response is biased away from the preferred orientation of the neurons that drive the lateral inhibition. Analogous effects of orientation-specific surround suppression on blood oxygen level dependent fMRI responses have been demonstrated in human V1, secondary visual cortex V2, and extrastriate visual cortical areas V3 and V4 (McDonald et al., 2009). Moreover, the tilt illusion exhibits considerable (80%) interocular transfer under dichoptic viewing conditions (Forte and Clifford, 2005), which implies a substantial cortical contribution. However, although V1 is the earliest stage of the primate visual pathway where orientation-tuning of neuronal responses is found (Hubel and Wiesel, 1968), orientation selectivity partly arises from significant biases already present at the LGN (Vidyasagar et al., 2015). Hence, there is the potential for additional pre-cortical contributions to contextual modulation of orientation.

In recent years, perceptual contextual effects in vision have been widely used to study a range of human conditions as an assay of the cortical balance between excitatory and inhibitory processes. An example of this is the study of healthy normal aging, where such tasks have been applied to indirectly study the presumed effects of altered cortical inhibition on visual processing. Older adults show stronger suppression of contrast for foveal viewing under a wide range of stimulus conditions, including variations of center–surround contrast (Karas and McKendrick, 2012, 2015), spatial phase (Karas and McKendrick, 2011), orientation (Karas and McKendrick, 2009), and stimulus duration (Karas and McKendrick, 2015). Here, we investigate the generality (or otherwise) of age-related effects on center–surround perceptual tasks, and test the hypothesis that the increase in perceptual surround suppression of contrast previously observed in older adults would similarly extend to other visual dimensions. Specifically, we chose tasks involving judgments of luminance, flicker, contrast, and orientation, in order to assess a range of fundamental visual stimulus properties, and to include tasks where the primary neural computations are considered to arise pre-cortically (luminance and flicker) or cortically (contrast and orientation). We were interested in whether the healthy aging process results in consistent or selective alterations to contextual processes across the visual dimensions, and whether performance is correlated between tasks. Such information not only enhances knowledge of the effects of aging on visual processing, but also provides information regarding the commonality of neural mechanisms responsible for these perceptual phenomena.

## Materials and Methods

### Participants

Two groups of observers were recruited to the study: 18 younger adults (12 females, aged 19–31, mean ± standard deviation: 24 ± 4 years) and 18 older adults (10 females, aged 60–75, 69 ± 5 years). A power analysis was performed using data from previous work that reported significantly increased perceptual surround suppression of contrast in older observers, relative to a younger cohort (Karas and McKendrick, 2012). The analysis indicated that 10 participants in each group provided a power of 0.95 (α = 0.05) for detecting a mean increase of 80% in suppression (large effect size: Cohen’s *d* = 1.75) in older observers relative to younger observers.

Ethics approval was granted by the Human Research Ethics Committee of the University of Melbourne. All participants provided written consent prior to participation in compliance with the tenets of the Declaration of Helsinki. An eye examination was conducted (refraction, ophthalmoscopy, slit lamp examination) to ensure the following eligibility criteria were met: best corrected visual acuity at least 6/7.5 or better with a refractive error between ± 5.00 D sphere and less than –2 D astigmatism, normal ocular health, no significant lens opacities defined as Grade 1.5 or less on the Lens Opacities Classification System III scale (Chylack et al., 1993), and no systemic conditions (e.g., diabetes, epilepsy) or medications (e.g., antidepressants) known to affect visual or cognitive function.

### Apparatus

The experimental software was written in Matlab V7.6 (Mathworks, Natick, MA, USA), with experimental stimuli presented on a gamma-corrected Sony G500 CRT monitor (Sony, Minato, Tokyo, Japan) using a ViSaGe graphics system (Cambridge Research Systems, Kent, UK). Participants were refractively corrected for the 80 cm working distance. Stimuli were viewed binocularly. The background was a homogenous gray screen of 50 cd/m^2^ mean luminance.

### Experimental Procedure

**FIGURE 1:**
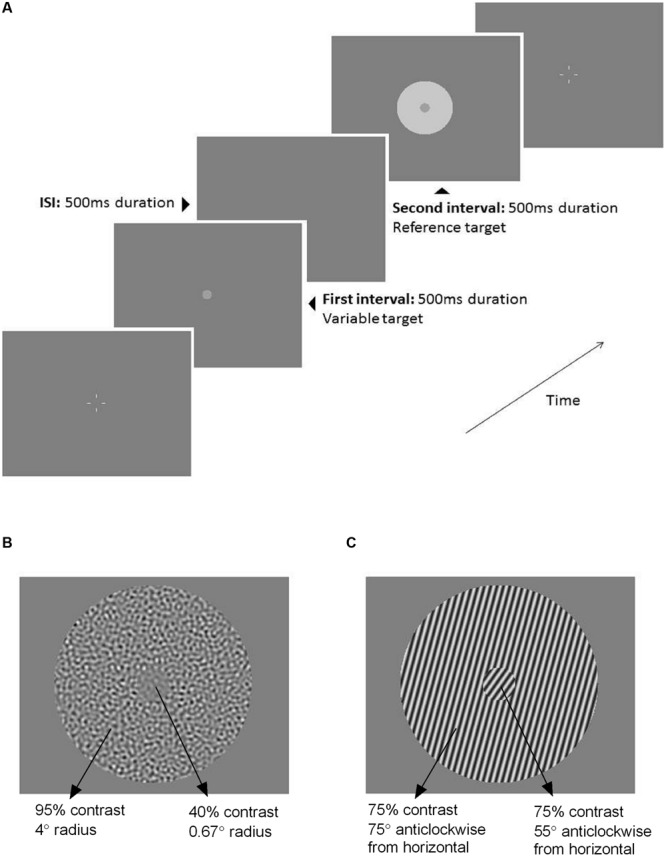
**(A)** Schematic of the two-interval forced choice (2IFC) procedure used throughout the testing. The first interval (500 ms) contained the variable target (0.67° radius) with no surround. The second interval (500 ms) contained the fixed, reference target that was either presented without a surround (‘no surround’ condition), or with a surround as depicted here (‘surround’ condition, 4° radius annulus). The interstimulus interval was 500 ms. Four white nonius lines appeared before and after each trial to assist with central fixation. The stimulus shown here is an example of the luminance task. Example ‘surround’ stimuli for the **(B)** contrast task and **(C)** orientation task.

Participants typically required 2 h to complete all of the tasks including regular rest breaks. There were eight tasks in total: four stimuli (luminance, flicker, contrast, and orientation) and two conditions (‘surround’ and ‘no surround’). The ‘no surround’ condition (0.67° radius center stimulus only) was tested first, followed by the ‘surround’ condition (center + 4° radius surround). The inclusion of a ‘no surround’ condition was important to establish that participants were able to accurately judge the stimuli (the specific judgments required are described below) and to account for any baseline biases. The order of the four stimuli was randomized for every participant and counterbalanced between older and younger groups to avoid order-dependent effects of learning or fatigue. Each task was tested twice using a Method of Constant Stimuli (MOCS) consisting of seven stimulus levels of 10 repeats (total 140 presentations per condition). For training purposes and to decide which stimulus levels to formally test for the ‘surround’ conditions, an initial abbreviated MOCS was performed (10 levels, two trials each). On each trial, participants viewed two stimuli (500 ms duration each) that were presented one after the other (two-interval forced choice, 2IFC) separated by a 500 ms interstimulus interval (**Figure 1A**). All stimuli shown were supra-threshold. Fixation was assisted by four white nonius lines, which appeared after each trial and disappeared during stimulus presentations.

### Luminance Task

For the luminance task, the monitor was configured to run at a frame rate of 100 Hz and have a resolution of 1024 × 768 pixels. Stimuli were homogenous circular patches, with the reference target of 55 cd/m^2^ central luminance and 72 cd/m^2^ surround luminance (**Figure 1A**). Participants were required to indicate the central stimulus (first or second interval) that appeared to be brighter by pressing one of two response buttons.

### Flicker Task

The monitor configuration was altered to allow for greater temporal resolution (frame rate 120 Hz, resolution 800 × 600 pixels). Similar to the luminance task, the flicker task involved homogenous circular patches except that the luminance contrast was temporally modulated. Both center and surround regions had a temporal contrast of 50% and were flickering at a rate of 15 Hz. To perceptually segment the center from the surround region, the two regions were presented with 180° phase difference given there was no gap between the center and the surround. The task was to choose which central stimulus appeared to be flickering with a greater depth of modulation.

### Contrast Task

The monitor configuration used for the contrast task was the same as for the luminance task (frame rate 100 Hz, 1024 × 768 pixel resolution). To avoid the potential confound of orientation information, the stimulus was a textured patch consisting of filtered noise (**Figure 1B**). The luminance noise images were constructed as per Denniss et al. (2014), i.e., bandpass filtered with a 1-octave width square-wave filter centered on 4 cycles/degree spatial frequency. The Michelson contrast of the reference center and surround was set at 40 and 95%, respectively. This contrast ratio between center and surround has consistently identified group differences in surround suppression between older and younger observers when tested foveally (Karas and McKendrick, 2009, 2012). Participants chose whether the first or second interval contained the central textured patch of higher contrast.

### Orientation Task

To present high resolution images, the monitor was configured to run at a frame rate of 80 Hz and resolution of 1264 × 948 pixels. For the orientation task, circularly windowed gratings of 4 cycles/degree spatial frequency were presented (**Figure 1C**). The reference center was oriented at 55° and the surround was oriented at 75°, given that the repulsive tilt illusion is greatest when the center and surround orientation differs by 20° (Clifford, 2014). All angles were calculated anticlockwise from horizontal. To avoid the potential confound of contrast information, and to maximize the repulsive tilt illusion (Qiu et al., 2013), the center and surround gratings were presented at the same contrast (75% Michelson). Participants were required to nominate which central grating was tilted closer to vertical (90°).

### Data Analysis

Psychometric functions were estimated by fitting a modified cumulative Gaussian (Eq. 1; Wichmann and Hill, 2001) using Microsoft Excel (Microsoft, Redmond, WA, USA).

(1) Ψ(t)=FP+(1−FP−FN)×G(t,μ,σ)

where *G(t*,μ,σ) is the cumulative Gaussian distribution with mean μ and standard deviation σ for value *t*. *FP* and *FN* represent the false positive and false negative error rates, respectively, assuming that false responses are made independently of the underlying Gaussian distribution of responses. Two parameters of interest were extracted for statistical analysis: (a) the mean of the fitted psychometric function (μ) or the ‘point of subjective equality’ (PSE), when both the reference and target stimulus appeared subjectively the same, and (b) the spread of the Gaussian (σ), which provides an estimate of precision, or the slope of the psychometric function. To quantify the strength of surround suppression for each task, a suppression index was calculated (1 – PSE ‘surround’/PSE ’no surround’). A positive suppression index indicates suppression (maximum suppression = 1), a negative index indicates facilitation, and an index of 0 indicates no effect of the surround.

### Statistical Analysis

**FIGURE 2:**
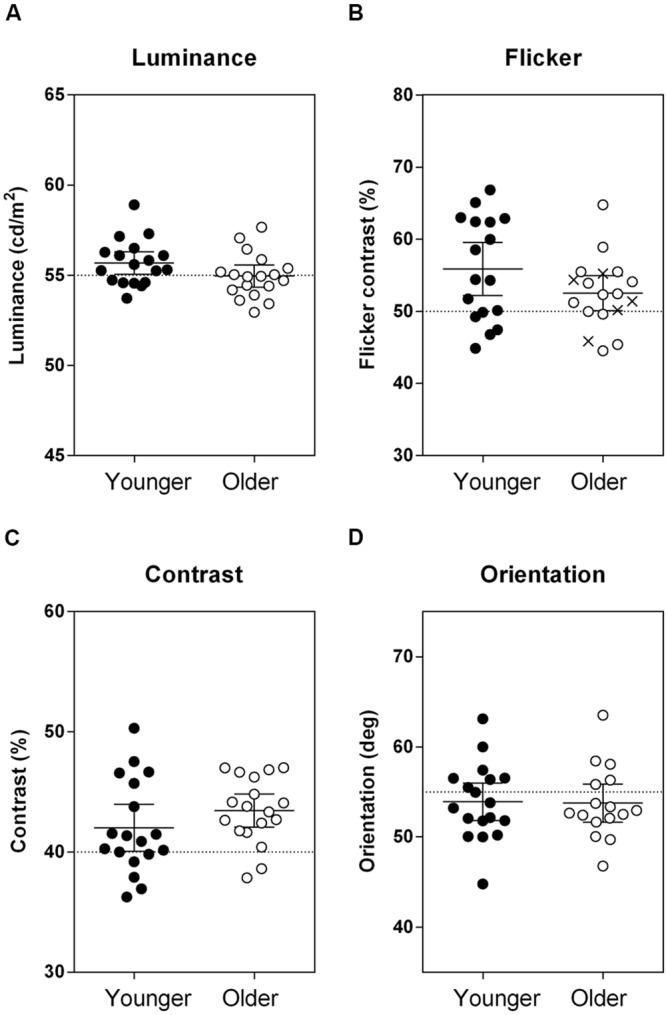
**Results from the four ‘no surround’ conditions, where the stimuli were defined by **(A)** luminance, **(B)** flicker, **(C)** contrast, and **(D)** orientation.** Group mean and individual data indicating the perceptual matches are plotted. The cross symbols in Panel **(B)** are the five individuals who could reliably match the depth of modulation of the flickering target with no surround, but could not perform the ‘surround’ version of the task because of a complete lack of flicker percept. Horizontal dotted lines indicate the veridical property of the target stimulus **(A)** 55 cd/m^2^, **(B)** 50% depth of modulation, **(C)** 40% contrast, and **(D)** 55° anticlockwise from horizontal meridian. Error bars are the 95% confidence limits of the mean. There was no overall difference in matching percepts between the groups across all of the tasks, *F*(1,34.66) = 0.79, *p* = 0.38.

Statistical comparisons were performed using SPSS Version 22.0 (SPSS Inc., Chicago, IL, USA). Data were tested to determine the probability that the sample was derived from a Gaussian distribution (Kolmogorov-Smirnov test). Three participants (one younger and two older observers) could not satisfactorily match one of the four ‘no surround’ percepts, whereas data were incomplete for the flicker suppression task for five older adults, who perceived zero flicker in the ‘surround’ condition, despite being able to reliably judge the depth of modulation in the ‘no surround’ condition (cross symbols in **Figure 2**). To avoid removing all data from a single observer if only one data point was missing, a linear mixed effect model was employed to compare older and younger group performance across all tasks. The fixed factors of the linear mixed effect model were ‘group’ (younger, older) and ‘stimulus’ (luminance, flicker, contrast, and orientation) and the random factor was ‘participant’ to control for non-independence among repeated observations for an individual.

## Results

A single mixed model analysis was used to first compare the performance of the groups when there was no surround (i.e., veridical perception). The PSE for older and younger observers did not differ for the baseline ‘no surround’ conditions [**Figure 2**; main effect of group: *F*(1,34.66) = 0.79, *p* = 0.38; group × stimulus interaction: *F*(3,100.63) = 2.42, *p* = 0.07]. This shows that both older and younger participants approximately matched to the same percept when there was no surround. Moreover, the spread of the psychometric functions (precision, or σ) was not different between groups [main effect of group: *F*(1,33.47) = 0.002, *p* = 0.97; group × stimulus interaction: *F*(3,99.88) = 0.73, *p* = 0.54] confirming no between-group difference in the precision with which stimulus comparisons were made.

**FIGURE 3:**
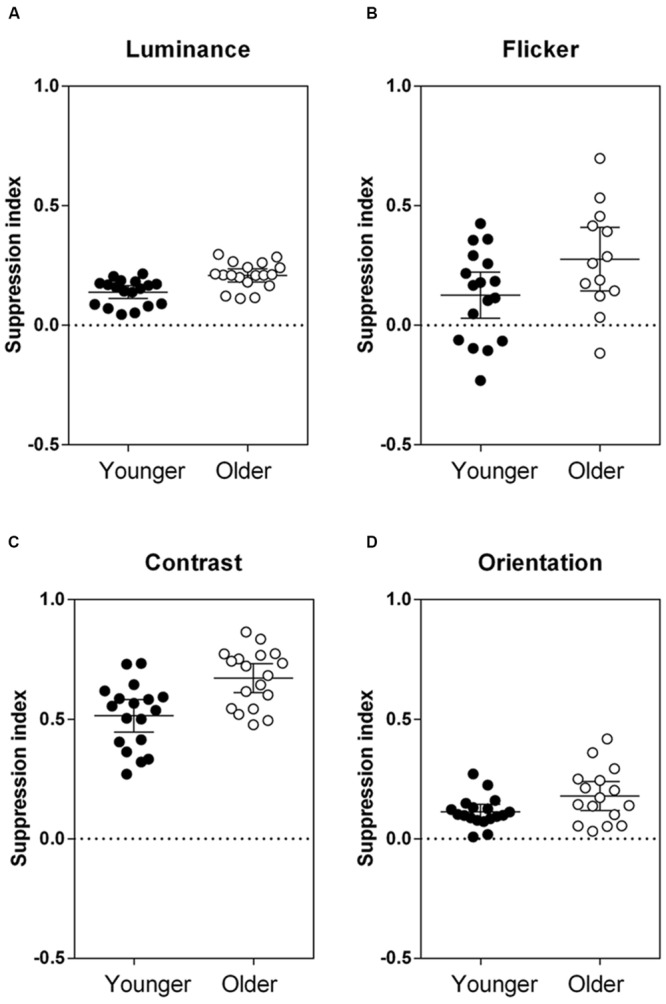
**Results from the four ‘surround’ conditions, where center–surround stimuli were defined by **(A)** luminance, **(B)** flicker, **(C)** contrast, and **(D)** orientation.** Group mean and individual suppression indices (1 – PSE ‘surround’/PSE ‘no surround’) are plotted, where a positive suppression index indicates suppression, a negative index indicates facilitation, and an index of 0 indicates no effect of the surround. Error bars are the 95% confidence limits of the mean. PSE, point of subjective equality. The two groups were significantly different from each other across all tasks, *F*(1,36.40) = 22.02, *p* < 0.001.

**FIGURE 4:**
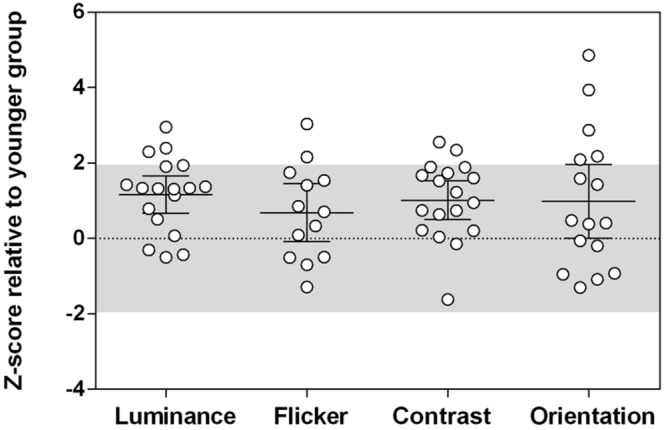
**The magnitude of contextual effects across different tasks in older observers.** *Z*-scores were calculated relative to the younger group performance. The shaded area is where 95% of the younger participants’ data lies (1.96 standard deviations from the mean). Positive *z*-scores indicate stronger suppression relative to the younger group mean, taking into account task variability. Error bars are the 95% confidence limits of the mean. There was no group difference in *Z*-scores across all tasks, *F*(3,98.15) = 0.23, *p* = 0.87.

Next, we used a separate mixed model analysis to compare the effect of introducing a surround by calculating suppression indices, as illustrated in **Figure 3**. The higher the suppression index, the greater the strength of suppression. The older group showed increased suppression relative to the younger participants for all stimuli [main effect of group: *F*(1,36.40) = 22.02, *p* < 0.001]. The difference in group performance was consistent across stimuli [group × stimulus interaction: *F*(3,99.17) = 1.37, *p* = 0.26]. Effect sizes (Cohen’s *d*, Eq. 2) were calculated to compare the magnitude of increased suppression in the older group, relative to the control group:

(2) $$d=(\mu_{1}-\mu_{2})/\sigma_{\mathrm{pooled}}$$

where,

(3) $$\sigma_{\mathrm{pooled}}\;=\;\sqrt{\frac{\sigma_{1}^{2}+\sigma_{2}^{2}}{2}}$$

and μ_1_ and μ_2_ are the mean suppression indices for the younger and older groups, respectively, and σ_1_ and σ_2_ are the standard deviations. Effect sizes were medium-large (Cohen’s *d* > 0.5) for all stimuli tested (luminance: *d* = 1.30, flicker: *d* = 0.73, contrast: *d* = 1.22, orientation: *d* = 0.72). To take into account differences in task variability, suppression indices were normalized against the performance of the control group (*z*-scores; **Figure 4**). The relative increase in surround suppression in older adults (increased positive *z*-score) was not stimulus-dependent [group × stimulus interaction: *F*(3,98.15) = 0.23, *p* = 0.87]. Thus, differences in contextual effects between older and younger observers appear to be widespread and of similar magnitude (medium–large effect size) across different visual dimensions.

**FIGURE 5:**
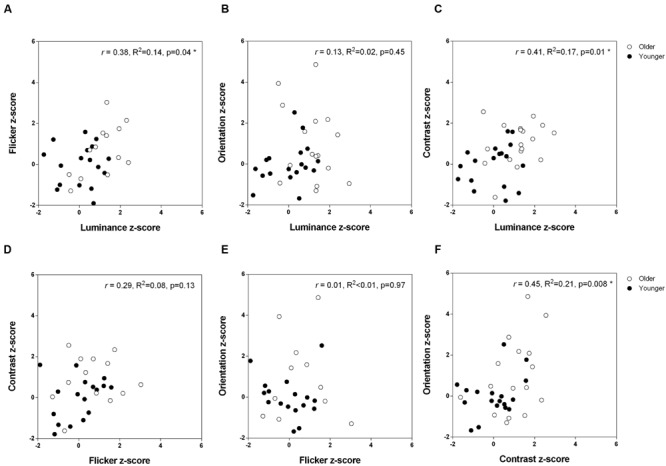
**The relationship between strength of surround suppression across the different tasks.** Pearson correlation coefficients are shown for the two groups combined. **(A)** Flicker versus luminance **(B)** Orientation versus luminance **(C)** Contrast versus luminance **(D)** Contrast versus flicker **(E)** Orientation versus flicker **(F)** Orientation versus contrast.

Does stronger suppression on one task predict stronger suppression on other tasks? Given that visual contextual performance is relatively uniform within each age group (younger versus older), we pooled the data from the two groups to obtain a range of suppressive strengths in the presence of a surround. **Figure 5** depicts the inter-task Pearson correlation analyses across the entire dataset based on the suppression indices, which takes into consideration baseline (no surround) performance. Statistical significance was defined at an alpha level starting at 0.008 (α_1_, Holm–Bonferroni correction), given there were six multiple comparisons. Under this criterion, we found a statistically significant positive correlation between contrast and orientation suppression (**Figure 5F**; Pearson *r* = 0.45, *R*^2^ = 0.21, *p* = 0.0076). Similarly, although these did not reach statistical significance based on sequential multiple comparisons (α_2_ = 0.01, α_3_ = 0.013), trends for a positive relationship between luminance and contrast suppression (**Figure 5C**; Pearson *r* = 0.41, *R*^2^ = 0.17, *p* = 0.014), and luminance and flicker suppression (**Figure 5A**; Pearson *r* = 0.38, *R*^2^ = 0.14, *p* = 0.038), were also observed. **Table 1** shows the correlation analyses for each group (younger and older) separately. None of these reached statistical significance once the 12 multiple comparisons were considered.

**Table 1:** Inter-task Pearson correlation analyses on suppression index *z-*scores for the two groups separately (older = shaded).

	Luminance	Flicker	Contrast	Orientation
Luminance		*r* = 0.01,	*r* = 0.19,	*r* = 0.31,
		*p* = 0.97,	*p* = 0.45,	*p* = 0.21,
		*R*^2^ < 0.01	*R*^2^ = 0.04	*R*^2^ = 0.09
Flicker	*r* = 0.57,		*r* = 0.27,	*r* = –0.16,
	*p* = 0.04,		*p* = 0.29,	*p* = 0.53,
	*R*^2^ = 0.32		*R*^2^ = 0.07	*R*^2^ = 0.03
Contrast	*r* = 0.26,	*r* = 0.12,		*r* = 0.34,
	*p* = 0.30,	*p* = 0.69,		*p* = 0.16,
	*R*^2^ = 0.07	*R*^2^ = 0.01		*R*^2^ = 0.12
Orientation	*r* = –0.25, *p* = 0.34, *R*^2^ = 0.06	*r* = –0.02, *p* = 0.95, *R*^2^ < 0.01	*r* = 0.39, *p* = 0.14, *R*^2^ = 0.15

*None of these reached statistical significance once the 12 multiple comparisons were considered (Holm-Bonferroni correction).*

## Discussion

We confirm that suppressive contextual effects on the perception of luminance, flicker, contrast, and orientation can be demonstrated in healthy individuals (Tibber et al., 2013; Yang et al., 2013a,b); however, on average, the magnitudes of these effects differ between older and younger observers. Despite ably matching the ‘no surround’ stimuli as well as their younger counterparts (**Figure 2**), as a group older participants showed a consistent increase in surround suppression for all targets tested (**Figure 3**). Our findings are congruous with previous observations that foveal suppression of perceived contrast is increased in older adults (Karas and McKendrick, 2009, 2011, 2012, 2015). Likewise, consistent with an earlier report (McCarter and Atkeson, 1977), the perceived brightness of a central patch was more affected in older adults by a surrounding light background than in younger adults (i.e., increased simultaneous brightness contrast effect). To our knowledge, we demonstrate for the first time that altered center–surround processing with normal aging is not confined to stimuli defined by contrast and luminance, but extends to other visual dimensions – flicker and orientation – that are presumed to rely on processing prior to, and at the level of, V1.

In the past decade or so, there has been considerable interest in investigating contextual effects in vision using perceptual center–surround stimuli to indirectly measure neuronal inhibitory/excitatory balance in the human visual system. Notwithstanding higher-level involvement (Mareschal and Clifford, 2012), both pre-cortical and cortical visual areas have been implicated as likely neural processing sites underlying such contextual effects. By implementing a battery of tests to assess center–surround processing at pre-cortical (luminance and flicker) and cortical (contrast and orientation) sites, we have considered whether the healthy aging process results in generalized or selective alteration to the neuronal circuitry responsible for surround suppression. In this study, increased surround suppression with aging was present for all stimuli, with no predilection for one attribute or another to show greater age-related effects once task variability was accounted for (**Figure 4**). Hence, age-related changes to contextual processing are widespread in the visual system. Our results are in agreement with two previous reports of greater age-related surround suppressive effects for luminance stimuli (McCarter and Atkeson, 1977) and low contrast center stimuli (Karas and McKendrick, 2015), which imply that differences in perceptual surround suppression in older adults could arise early in the visual pathway (i.e., pre-cortically at the LGN or input layers of V1). Luminance suppression is presumed to arise pre-cortically via lateral inhibition in the retina and LGN (Valberg et al., 1985; Creutzfeldt et al., 1991), while contrast-dependent surround suppression is monocularly driven and broadly tuned for spatial and temporal frequency when the center contrast is low, implicating involvement of the LGN and V1 input layers (Webb et al., 2005). Experiments are underway in our laboratory to further investigate the neuronal bases of altered surround suppression with normal aging (using dichoptic methods, for example) to differentiate pre-cortical from cortical mechanisms.

The commonality of the increased suppressive effects for older adults across stimulus dimensions in our study does not imply that all perceptual measures of surround suppression show increased suppression with age. An alternate task for investigating surround suppressive effects in vision – the motion duration task described by Tadin et al. (2003) – reveals a different pattern of results. As the size of a high-contrast, drifting grating increases, the stimulus duration required to correctly identify the direction of motion becomes increasingly longer, which has been attributed to surround suppression in the motion processing visual area V5/MT (Tadin et al., 2003, 2011). Older adults show less, rather than more, suppression on this motion direction discrimination task (Betts et al., 2005, 2009; Yazdani et al., 2015). Two studies that have used variants of both a suppressive contrast task (where there is a clear boundary between the center and surround regions) and motion task (involving a single drifting grating patch) in the same older and younger individuals confirm a lack of concordance in the outcomes of these two measures of surround suppression across individuals (Karas and McKendrick, 2012; Yazdani et al., 2015). This finding has been argued as evidence for different mechanisms underpinning performance on contrast and motion suppression tasks. Similarly, while contextual modulation of foveal perceived contrast is age-dependent (McKendrick et al., 2013), surround suppression of parafoveal (4–5° eccentricity) contrast sensitivity remains constant between the ages of 20 and 70 when tested with a detection task (Serrano-Pedraza et al., 2014; Yazdani et al., 2015). This suggests that aging effects on surround suppression of contrast sensitivity and supra-threshold perceived contrast are also not equivalent – possibly owing to differences between foveal and parafoveal viewing – and that these two measures likely reflect independent neuronal mechanisms involved in the contextual processing of contrast.

By testing the same observers across all tasks, we could examine whether performance was correlated between tasks and hence, infer whether the neuronal mechanisms involved in the contextual processing of fundamental visual attributes are independent. Performance was significantly, albeit modestly, correlated between certain stimuli across our range of ages (**Figure 5**), providing indirect support for a common process underlying age-related differences in center–surround perception. Perhaps not surprisingly, the two center–surround stimuli presumed to rely predominantly on pre-cortical processing (luminance and flicker) were correlated, as were the tasks with principal V1 involvement (contrast and orientation). In addition, the significant correlation between the strength of perceptual suppression of contrast (cortical) and luminance (pre-cortical) is consistent with surround suppression at V1 being, at least partly, inherited from pre-cortical surround suppression at the retina and LGN. On the other hand, previous reports find no such correlations between different tests of surround suppression (luminance, size, contrast, orientation, and motion), and conclude that these tasks reflect distinct neural mechanisms (Tibber et al., 2013; Yang et al., 2013a,b). An important distinction between these previous studies (Tibber et al., 2013; Yang et al., 2013a,b) and ours is the patient group involved. Here, we tested normal, healthy observers who lie along an age continuum (19–75 years), whereas previous studies have looked for inter-task relationships within distinct clinical groups, i.e., schizophrenic, bipolar, and healthy controls (Tibber et al., 2013; Yang et al., 2013a,b). Furthermore, we were careful to address some methodological limitations of earlier work. For one, Yang et al. (2013a,b) presented stimuli until a response was made, which creates an additional source of variability given that surround suppressive effects can depend on stimulus duration. The magnitude of the repulsive tilt illusion increases for durations up to 100 ms but then decreases thereafter (Calvert and Harris, 1988), while adaptation to the surround occurs when a high-contrast stimulus is presented for longer durations, thus rendering the surround less effective at suppressing the response of macaque V1 neurones (Cavanaugh et al., 2002; Patterson et al., 2013) and reducing the perceived contrast in human observers (e.g., 100 ms vs. 500 ms; Karas and McKendrick, 2015). Moreover, unlike the study by Tibber et al. (2013) where contextual effects were expressed relative to the veridical stimulus property, we measured the PSE for ‘no surround’ conditions to take into consideration inherent biases of participants (Yang et al., 2013a,b). Normal observers vary in their perception of the stimulus without it being embedded in the surround (**Figure 2**), possibly owing to button response bias and/or differences in masking/adaptation due to the 2IFC design. Given that the magnitude of contextual effects depends on the relative difference in percept once a surround is introduced, group differences in the strength of suppression may be less evident if the ‘no surround’ PSE is assumed to be a single constant that is common to all observers.

In interpreting our results, it is important to note that our small sample sizes were sufficient (based on our power analysis) to detect differences in suppression strength between older and younger observers (**Figure 3**), as evidenced by the medium–large effect sizes reported in this study, but that our correlational analysis (**Figure 5**) might only have reached statistical significance had more participants with complete data been tested. It should also be noted that our older participants may not be representative of all older adults – rather, they are healthy, lead active lives and live independently within the community.

We demonstrate that surround suppression of luminance, flicker, contrast, and orientation is increased in older adults, implying a generalized contextual processing change with normal aging. Further work is needed to disentangle the specific neural mechanisms involved; however, this study provides a basis for targeted behavioral and neurophysiological approaches to be applied to the study of the aging visual neural system.

## Author Contributions

Study design: BN and AM. Subjects and data collection: BN. Data analysis and interpretation: BN and AM. Paper drafting and revision: BN and AM. Final approval of the version to be published: BN and AM. Agreement to be accountable for all aspects of the work in ensuring that questions related to the accuracy or integrity of any part of the work are appropriately investigated and resolved: BN and AM.

## Conflict of Interest Statement

The authors declare that the research was conducted in the absence of any commercial or financial relationships that could be construed as a potential conflict of interest.
